# Results from 237 extracorporeal membrane oxygenation runs with drowned patients: a nationwide retrospective study

**DOI:** 10.1186/s13054-023-04580-w

**Published:** 2023-07-20

**Authors:** Thomas Jasny, Jan Kloka, Oliver Old, Florian Piekarski, Gösta Lotz, Kai Zacharowski, Benjamin Friedrichson

**Affiliations:** Goethe University Frankfurt, University Hospital, Department of Anaesthesiology, Intensive Care Medicine and Pain Therapy, Theodor-Stern Kai 7, 60590 Frankfurt am Main, Germany

**Keywords:** ECMO, Drowning, Nationwide, In-hospital mortality

## Abstract

**Background:**

Drowning is one of the leading causes of death worldwide and presents with a wide range of symptoms, from simple coughing to cardiac or pulmonary failure. In severe cases, extracorporeal membrane oxygenation (ECMO) should be considered as a rescue therapy. Therefore, we sought to analyse ECMO usage, outcomes and predictive factors in drowned patients.

**Methods:**

The Federal Statistical Office of Germany provided the study data. The patients included experienced drowning (ICD T75.1) and ECMO (OPS 8–852.0, 8–852.3) between 2007 and 2020. All age groups were included. Mortality was calculated for the total population and for ECMO patients. A multiple logistic regression model for ECMO patients was applied to account for predefined patient characteristics and complications.

**Results:**

Of 12,354 patients who were hospitalised due to drowning, 237 patients (1.9%) received ECMO. Hospital mortality was 14.1% (*n* = 1741) overall and 74.7% (*n* = 177) for ECMO patients. In-hospital mortality was positively associated with cardiopulmonary resuscitation (CPR) before admission (odds ratio [OR] 4.49, 1.31–15.39) and in-hospital CPR (OR 6.28, 2.76–14.31). Stroke (OR 0.14, 0.02–0.96) and drug abuse (OR 0.05, 0.01–0.45) were negatively associated with in-hospital mortality. Neither the ECMO mode nor the patient’s age and sex had statistically significant effects on survival.

**Conclusion:**

This study indicates that survival in drowned patients who receive ECMO is lower than previously reported. The proportion of paediatric patients was also smaller than expected. As the effects of different ECMO modes on mortality remain unclear, the need for further study remains great.

## Background

Drowning is one of the leading causes of death worldwide, with an estimated 236,000 deaths in 2019, excluding intentional deaths, floods and traffic accidents [1]. Drowned and submerged people present a wide range of symptoms. In mild cases, these symptoms include simple coughing and troubled breathing; severe cases of drowning can lead to cardiac and pulmonary failure [2]. Especially in severe cases, advanced resuscitation interventions are limited. The European Resuscitation Council guidelines state that extracorporeal membrane oxygenation (ECMO) should be considered as a rescue therapy to prevent or treat cardiac arrest as a direct result of drowning [3]. ECMO can also be applied to treat the secondary consequences of drowning, e.g. oedema or pneumonia due to aspiration that develops into acute respiratory failure (ARDS) [4]. ECMO has become a vital instrument in modern intensive care medicine. Its indications are various cardiac and pulmonary conditions, including cardiac and pulmonary failure [4, 5]. Despite increasing experience using ECMO, protocol improvements and equipment advances, it remains an invasive form of rescue therapy that may result in various complications. These complications include major bleeding, acute kidney injury, new infections and acute neurologic events [6–10]. Although drowning is a common cause of accidental death, studies examining the usage of ECMO usage in these patients remain urgently needed [11]. Many of the existing studies are small, single-centre studies, with case numbers that rarely exceed 15 patients [12].

This study sought to analyse the usage of ECMO for all patients with cardiac and pulmonary failure due to drowning or submersion in Germany from 2007 to 2020. In this nationwide study, the hospital mortality rate for drowned and submerged patients regardless of therapy was analysed and the predictive factors for patients receiving ECMO were examined.

## Materials and methods

The Federal Statistical Office of Germany provided data from 2007 to 2020 [13]. All hospitals in Germany are legally required to report every inpatient for reimbursement and to develop the diagnosis-related group system. The retrospective secondary data included diagnoses encoded using the international statistical classification of diseases and related health problems (ICD) system and the procedures performed using process keys (OPS). Due to the Federal Statistical Office’s institutional anonymisation, the Ethics Committee of the University Hospital Frankfurt waived the need for ethical committee approval (Chair: Prof Dr Harder, Ref: 2022-766). The data were collected in a structured and representative manner according to the Declaration of Helsinki.

All patients who had experienced drowning or nonfatal submersion (ICD T75.1) and ECMO support were included, regardless of age. We differentiated between venovenous (V-V) ECMO (OPS 8–852.0) and venoarterial (V-A) ECMO (OPS 8–852.3). Due to the age grouping of the Federal Statistical Office of Germany, paediatric patients were defined as patients aged up to and including 19 years.

The data were analysed descriptively and inferentially. The primary outcome was in-hospital mortality, which was calculated for all patients and patients who received (specific) ECMO therapies. Categorical variables were shown as absolute numbers and percentages. As all continuous variables were non-normally distributed, we displayed the 25%, 50% (median) and 75% quartiles. The differences between the ECMO groups (survivors and non-survivors) for predefined comorbidities, complications and Elixhauser scores, using their respective ICD and OPS codes [14, 15], were estimated with the Wilcoxon rank-sum test for continuous variables and with a chi-squared test for categorical variables. The significance level was set to 5%. Obesity was defined as a body mass index (BMI) greater than 30 (WHO Grade I). A multiple logistic regression model for patients who received ECMO was applied to adjust for age, sex, V-A ECMO, V-V ECMO, intracranial bleeding, stroke, pulmonary embolism, embolism and thrombosis, myocardial infarction, cardiopulmonary resuscitation (CPR) before admission, in-hospital CPR, dialysis, congestive heart failure, hypertension, chronic pulmonary disease, diabetes, renal failure, obesity, liver disease and drug abuse. SAS (Version 9.4M6, SAS Institute Inc., Cary, NC, USA) was used for the statistical analysis, and figures were created using RStudio (Version 2022.12.0 + 353, Posit PBC, Boston, MA, USA).

## Results

**Fig. 1:**
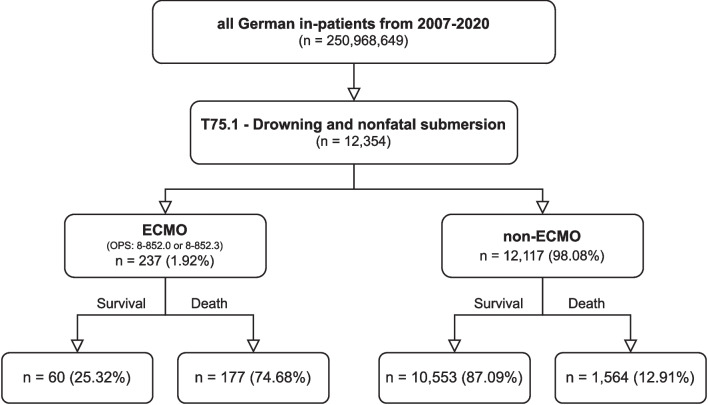
Patient flow-chart

In total, 12,354 patients were hospitalised due to drowning and nonfatal submersion from 2007 to 2020. Of these, 237 patients (1.9%) received ECMO support. Hospital mortality was 14.1% (*n* = 1741) overall and 74.7% (*n* = 177) for patients who received ECMO (Fig. 1).

### Annual case volume

**Fig. 2:**
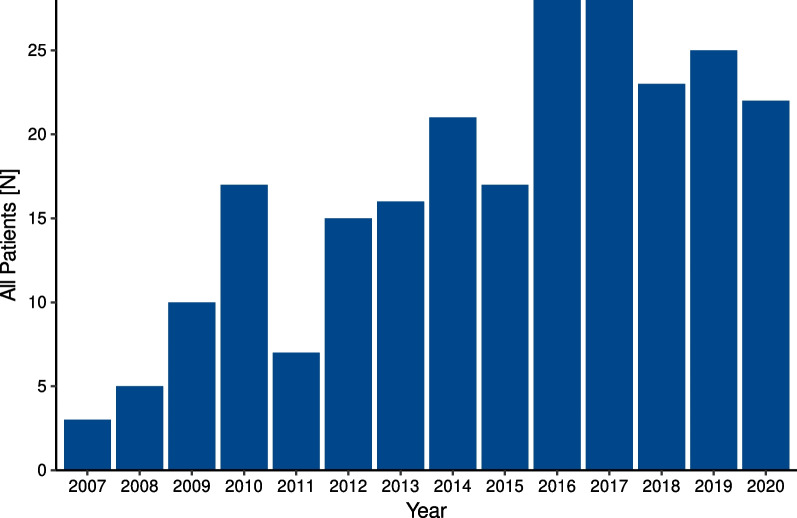
Annual number of drowned patients receiving ECMO for the years 2007 to 2020

The average annual case volume was 882.4 (SD = 108.3) per year overall and 16.9 (SD = 8.2) for ECMO patients. ECMO usage increased from 0.3% (*n* = 3) of patients in 2007 to 2.7% (*n* = 22) in 2020, with maximums of 2.8% and 2.9% *(n* = 28) in 2016 and 2017, respectively (Fig. 2).

### Patient characteristics

There were more male than female ECMO patients, with 74.7% (*n* = 177) male and 25.3% (*n* = 60) female patients. Survival rate did not differ significantly (*p* = 0.45) by sex when analysed. The median hospital stay for patients in the ECMO group was significantly longer (*p* < 0.01) for survivors at 24.8 d (16.3, 31.9). For non-survivors, the median hospital stay was 0.9 d (0.3, 3.1).

ECMO modes were significantly associated with survival (*p* < 0.01), with a survival rate of 37.7% (*n* = 43) for V-V ECMO and 13.8% (*n* = 17) for V-A ECMO (Table 1).

**Table 1:** Patient characteristics for drowned patients receiving ECMO

		Survivors			Non-survivors		
		*N*	%		*N*	%	*p* value
Total		60			177		
Female		13	21.67		47	26.55	0.4518
V-V ECMO		43	71.67		71	40.11	< 0.0001
V-A ECMO		17	28.33		106	59.89	< 0.0001

	Q1	Median	Q3	Q1	Median	Q3	*p* value
Age [yr]	16.50	29.00	39.00	14.00	29.00	50.00	0.6008
Hospital stay [d]	16.30	24.82	31.88	0.26	0.86	3.07	< 0.0001
Elixhauser score	5.00	9.00	14.50	5.00	11.00	16.00	0.3572

		*N*	%		*N*	%	*p* value
Comorbidites							
Congestive heart failure		10	16.67		25	14.12	0.6314
Hypertension		8	13.33		7	3.95	0.0099
Chronic pulmonary disease		*	*		*	*	0.0183
Diabetes		*	*		*	*	*
Renal failure		4	6.67		5	2.82	0.1785
Obesity		*	*		*	*	*
Liver disease		6	10.00		33	18.64	< 0.0001
Drug abuse		*	*		*	*	*
Complications							
Intracranial Bleeding		*	*		*	*	0.6216
Stroke		5	8.33		5	2.82	0.0666
Pulmonary embolism		*	*		*	*	*
Arterial embolism and/or thrombosis		*	*		*	*	0.6474
Myocardial infarction		0	0.00		3	1.69	0.3102
CPR prior to admission		7	11.67		42	23.73	0.0462
In-hospital CPR		13	21.67		108	61.02	< 0.0001
Dialysis		19	31.67		41	23.16	0.1905

Legend: V-V—venovenous; V-A—venoarterial; ECMO—extracorporeal membrane oxygenation; CPR—cardiopulmonary resuscitation

*Censored

### Age distribution

In the ECMO group, the ages of survivors and non-survivors did not differ significantly (*p* = 0.60). Survivors’ median age was 29 years (16.5, 39.0) and non-survivors’ was 29 years (14.0, 50.0).

**Fig. 3:**
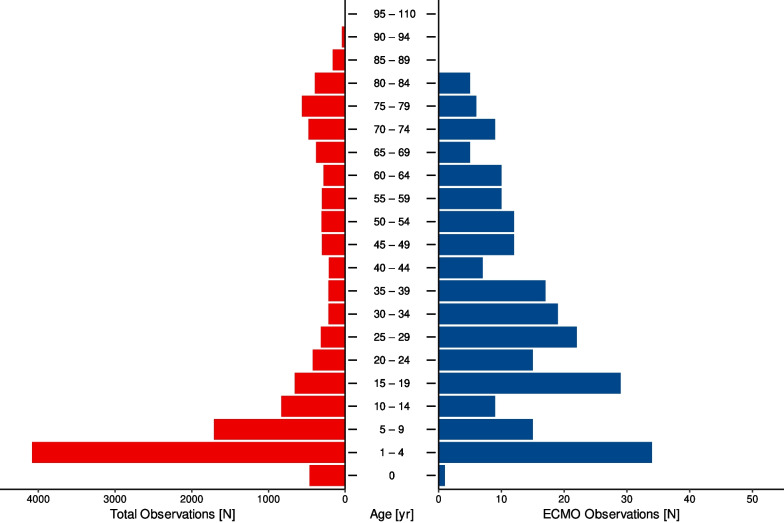
Age distribution for drowned patients. Total population in red, ECMO population in blue

The age distribution for all patients hospitalised due to drowning and submersion was bimodal, with an increased incidence among children aged 1–4 years. A second, lower peak was seen among patients aged 75–79 years. For patients who received ECMO, the age distribution was positively skewed. Paediatric patients (up to and including 19-year-olds) comprised 37.1% (*n* = 88) of this population, and adolescent patients (older than 19 years) comprised 62.9% (*n* = 149) (Fig. 3).

### Predictive factors

**Fig. 4:**
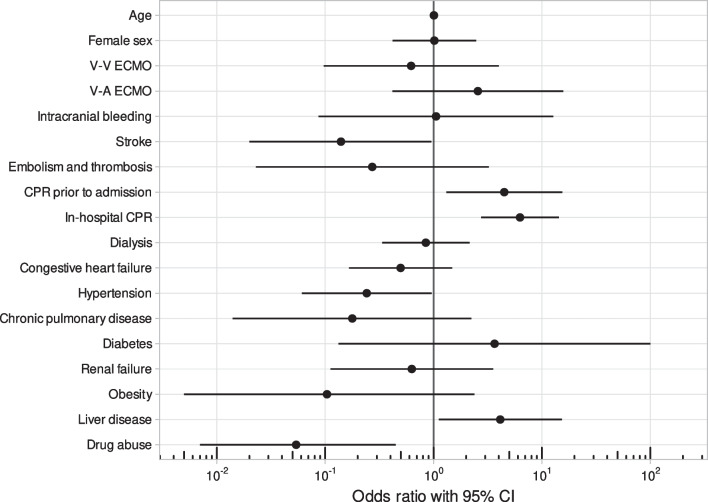
Odds ratio for drowned patients receiving ECMO with mortality as outcome. *Legend*: V-V—venovenous; V-A—venoarterial; ECMO—extracorporeal membrane oxygenation; CPR—cardiopulmonary resuscitation

Multiple logistic regression showed that the probability of in-hospital mortality was positively associated with CPR before admission, with an odds ratio (OR) of 4.49 (1.31–15.39). In-hospital CPR had an OR of 6.28 (2.76–14.31), and liver disease had an OR of 4.13 (1.12–15.25). The probability for in-hospital mortality was negatively associated with stroke and drug abuse, with ORs of 0.14 (0.02–0.96) and 0.05 (0.01–0.45), respectively. The ECMO mode did not have a statistically significant effect on mortality, with an OR of 0.62 (0.10–3.99) for V-V ECMO and 2.57 (0.42–15.74) for V-A ECMO. Age (OR 1.01, 0.99–1.03) and sex (OR 1.01, 0.42–2.47) were also not found to have statistically significant impacts on mortality (Fig. 4).

## Discussion

This study is the first nationwide retrospective analysis of ECMO usage for drowned and non-fatally submerged patients, with a total of 237 ECMO runs. The survival rate of drowned patients who received ECMO support was lower than reported in previous studies.

### Comparison of mortality

A comparison of our findings with those of previous studies was difficult because the existing studies were single-centre studies with small samples that rarely exceeded 15 individuals [12]. Burke et al. used retrospective data from the Extracorporeal Life Support Organisation (ELSO) registry and analysed 247 cases from 1986 to 2015 [16]. The reported survival rate of 51.4% *(n* = 127) was considerably higher than our findings of a 25.3% *(n* = 60) survival rate [16]. When comparing these results, a few important aspects should be considered.

First, patient data in the ELSO registry are collected internationally. Therefore, there are different definitions for drowning and submersion, as well as differences in their pathophysiologic backgrounds [2, 17]. For example, drowning in a cold sea can lead to additional hypothermia, whereas drowning in a warm whirlpool cannot. The clinical approaches and outcomes for these patients are, therefore, influenced by the type of drowning experienced [18]. Different parts of the world are also likely to have different incidences of specific types of drowning, requiring different clinical approaches and, therefore, resulting in different survival rates. A distinction between these types would be necessary for a better comparison, but the ICD-10 system and, therefore, the data source for this study does not permit such a distinction. Second, centres submitting data to the ELSO registry are most likely experienced, while many low-volume centres exist in Germany as ECMO usage is unregulated [19]. Third, the ELSO registry is based on data that contributing centres send voluntarily [20]. The contribution of hospitalisation data to the Federal Statistical Office of Germany is legally required, decreasing the risk of bias in analyses of these data registries. By using data from every hospitalisation for drowning in Germany, we obtained a more realistic overview of patient mortality without having to adjust for expertise bias.

### Age distribution

The age distribution for all patients receiving ECMO due to drowning was positively skewed, with paediatric patients comprising 37.1% (*n* = 88) of the total. The age distribution for drowned patients regardless of the therapy they received was multimodal, with a major mode for young children aged 1–4 years, followed by a steep decrease in incidence for older children and a much smaller peak for young adolescents [21]. We found a similar distribution when examining all patients. When examining ECMO usage, paediatric patients comprised a much lower portion of the population, meaning that paediatric patients are less likely to receive ECMO support than adolescent patients. This is surprising as ECMO tends to have better outcomes for paediatric patients [22]. This result could reflect that children are more likely to be sent to the hospital for observation in mild cases of drowning as people tend to be more cautious when treating children. This would result in mild cases comprising a larger proportion of the patients in these age groups compared to adolescents and could explain the disparity. Another reason could be that paediatric ECMO centres are located almost exclusively at large tertiary-care hospitals and are, therefore, not broadly available.

In contrast, Burke et al. found that paediatric patients comprised 80.2% (*n* = 198) of the patients in an analysis of the ELSO registry [16]. The method of data aggregation in that study must be considered as, of the over 400 contributing ELSO centres, 350 were paediatric centres. This bias of a registry could have led to such a shift in the results and underlines the importance of having a complete data set of a whole country.

Age was not significantly associated with survival, which aligns with previous studies on drowning [16]. However, ECMO support tends to have better outcomes for paediatric patients in other use cases [22]. In our study age was distributed multimodally, therefore a sub-analysis on drowned paediatric and adolescent patients might reveal further insights.

### The mode of extracorporeal membrane oxidation

When descriptively analysed, both ECMO modes played significant roles (*p* < 0.01) in survival. This also aligns with previous studies [16]. The main indication for the use of V-V ECMO is respiratory failure, while V-A ECMO is used for patients who are experiencing cardiopulmonary failure [23]. Therefore, a higher mortality rate for patients who received V-A ECMO seems logical. When a multiple logistic regression model was applied, V-A ECMO trended towards mortality (OR 2.57, 0.42–15.74), while V-V ECMO trended towards survival (OR 0.62, 0.10–3.99). However, neither mode was significant. An explanation for this trend could be the usage of ECMO in the form of extracorporeal cardiopulmonary resuscitation (ECPR), which is known to be associated with increased mortality when compared to normal ECMO support. Unfortunately, this dataset does not allow for a distinction between ECPR and ECMO implantation after CPR.

### Limitations

Correct documentation is incentivised as it directly affects hospital reimbursement. However, these data do not provide any information on laboratory values or the drowning events, such as immersion time. The data only provide information on in-hospital and non-pre-hospital factors that are likely to affect survival. Due to limitations of the coding classifications and due to rules regarding data protection, the dataset does not allow for an inclusion of all common drowning complications in our analysis, e.g. neurologic injuries. Furthermore, there is no specific coding for ECPR.

## Conclusion

This study indicates that survival in drowned and non-fatally submerged patients who receive ECMO is lower than previously reported. The proportion of paediatric patients was also smaller than expected based on previous studies, and the effects of different ECMO modes on mortality remain unclear. Further investigation is necessary to evaluate outcomes on a centre level and differentiate the outcomes of different types of submersion. Due to the low application of ECMO to drowned patients, there is still a great need for studies on this topic.

## Data Availability

The Federal Statistical Office of Germany provided all data used. According to $21KHEntgG, reimbursement data are free for scientific use, but its availability is restricted and not publicly available. Upon reasonable request and with permission of the Federal Statistical Office of Germany, data are available from the authors. The data that support the findings of this study are available from the Federal Statistical Office of Germany but restrictions apply to the availability of these data, which were used under licence for the current study, and so are not publicly available. Data are however available from the authors upon reasonable request and with permission of the Federal Statistical Office of Germany.

## Abbreviations

ARDS: Acute respiratory distress syndrome
BMI: Body mass index
CPR: Cardiopulmonary resuscitation
ECMO: Extracorporeal membrane oxygenation
ECPR: Extracorporeal cardiopulmonary resuscitation
ELSO: Extracorporeal Life Support Organisation
ICD: International statistical classification of diseases and related health problems
OPS: Process keys
OR: Odds ratio
SD: Standard deviation
V-A: Venoarterial
V-V: Venovenous
